# Microbiome-driven alterations in tryptophan metabolism contribute to behavioral comorbidities in the Muc2 knockout mouse model of chronic colitis

**DOI:** 10.1080/19490976.2026.2689121

**Published:** 2026-06-24

**Authors:** Jessica K. Josephson, Jacqueline A. Barnett, Emad Yuzbashian, Andrea Verdugo-Meza, Susan J. Murch, Tyler J. Wenzel, Andis Klegeris, Keith A. Sharkey, Deanna L. Gibson

**Affiliations:** a Department of Biology, University of British Columbia, Kelowna, BC, Canada; b Department of Chemistry, University of British Columbia, Kelowna, BC, Canada; c Department of Psychiatry, University of Saskatchewan, Saskatoon, SK, Canada; d Department of Physiology and Pharmacology, Hotchkiss Brain Institute and Snyder Institute for Chronic Diseases, Cumming School of Medicine, University of Calgary, Calgary, AB, Canada; e Department of Medicine, Faculty of Medicine, University of British Columbia, Vancouver, BC, Canada

**Keywords:** Gut microbiome dysbiosis, gut–brain–microbiome axis, tryptophan, kynurenine, serotonin, colitis

## Abstract

Globally, the incidence of inflammatory bowel disease (IBD) is projected to reach 0.5% of the population by 2030, with increasing recognition of neurobehavioral comorbidities, including anxiety, depression, and cognitive dysfunction. The mechanisms underlying these comorbidities remain unclear but may involve interacting pathways, including microbial dysbiosis, inflammation and imbalanced neurometabolite production. Here, we investigated whether microbiome-associated alterations in neurometabolites are correlated with behavioral changes in a chronic colitis model. Specific pathogen-free (SPF) and germ-free (GF) mucin 2 knockout mice (Muc2^−/−^) alongside mucin 2 expressing mice (Muc2^+/+^) were evaluated for behavioral patterns of anxiety, depressive-like patterns and memory dysfunction. Tryptophan and metabolite concentrations were measured in the colon, serum and brain. Blood–brain barrier integrity and neuroimmune activation were assessed through tight-junction protein claudin-5 expression, glial fibrillary acid protein (GFAP) and ionized calcium-binding adaptor molecule 1 (IBA-1) protein expression. Microbiome composition was characterized in relation to the tryptophan utilization pathways. To assess causality, early-life nutrient supplementation was used to address potential metabolite depletion. Female Muc2^−/−^ displayed reduced anxiety-like behavior, while males displayed memory dysfunction. These changes coincided with decreased intestinal tryptophan, kynurenine, and serotonin within the gastrointestinal tract. GF Muc2^−/−^ mice displayed normalized intestinal metabolite levels without concurrent brain metabolite changes. Notably, behavioral phenotypes were lost in GF Muc2^−/−^ mice, revealing a key role for the microbiome played in these comorbidities. Muc2^−/−^ exhibited reduced claudin-5, suggesting impaired blood‒brain barrier integrity. Microbiome analysis revealed a shift towards indole production and NAD+ salvage pathways with reduced abundance of *Anaerotruncus*, *Enterocloster* and *Intestinimonas*. Although early-life nutrient supplementation partially restored colonic tryptophan, it failed to fully rescue behavioral outcomes. Collectively, these findings demonstrate that chronic colitis is associated with microbiome-mediated disruption of host tryptophan metabolism, which correlates with neurobehavioral dysfunction. Targeting microbiome-driven metabolic alterations may represent a therapeutic strategy for both intestinal and neurobehavioral manifestations of IBD.

## Graphical abstract

**Figure:**
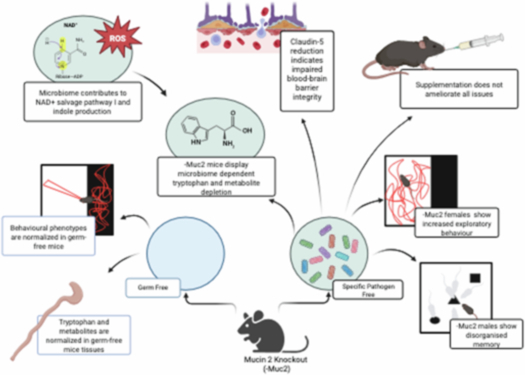

## Introduction

Chronic inflammatory diseases such as inflammatory bowel disease (IBD) have been increasingly associated with neuropsychiatric comorbidities such as anxiety,^1^ ^,^ ^2^ depression^3^ ^,^ ^4^ and memory deficits.^5^ Epidemiological findings reveal that neurological diseases are co-morbid with IBD in up to 47.5% of patients.^6^ Of concern, co-morbid psychiatric conditions also correspond with more severe IBD symptoms.^2^ Subsequently, increased disease severity requires more drastic interventions for patients to manage their disease effectively.

The pathogenesis of behavioral comorbidities during colitis may result from disruptions in the bidirectional communication between the gastrointestinal (GI) tract, the brain, and the microbiome, known as the gut‒brain-microbiome axis. The pathophysiology of this relationship in colitis has yet to be fully elucidated, with perturbations attributed to various sources including microbiome dysbiosis,^7^ chronic inflammation,^8^ and imbalanced nutrient utilization.^9^ These effects differ depending on the duration of colitis with short-term models showing alterations in anxiety and depression-like behavioral profiles,^10^ ^,^ ^11^ and recurring colitis episodes increasing observed fear in murine models.^12^ For long-term and recurring dysbiosis and inflammation, the shift in metabolite production and nervous communication contributes to these comorbidities, however, there is some evidence of tolerogenic behavioral adaptations occurring over repeated cycles of gut inflammation.^13^

Tryptophan and its neurometabolites kynurenine and serotonin are influenced by inflammation present in both acute and chronic colitis.^14^ Recognized as a key gut–brain axis signaling pathway, tryptophan and its metabolites have diverse roles across the body, including in modulating immune responses,^15^ ^,^ ^16^ regulating gut motility and function^17^ and communicating with the central nervous system (CNS) through the enteric nervous system.^18^ ^,^ ^19^ Increasing evidence from animal studies suggests that tryptophan can be utilized by the gut microbiome.^18^ Indeed, a dysbiotic microbiome has the potential to over-utilize available luminal tryptophan. For example, *Lactobacilli* spp. are tryptophan-utilizing bacteria producing a variety of indole derivatives, which impact host immunity and intestinal homeostasis through aryl hydrocarbon receptor (AhR) activation.^19^ Dysregulation of tryptophan metabolism is known to contribute to the development of neuropsychiatric comorbidities,^18‐21^ with disruption of a portion of this pathway through the use of a serotonin agonist showing a reversal of anxiety and depressive-like behaviors in a colitis model.^22^ Chronic inflammation, unlike acute inflammation, results in a prolonged upregulation of the inflammatory kynurenine metabolism pathway,^14^ ^,^ ^23^ resulting in a depletion of tryptophan (increasing the kynurenine: tryptophan ratio).^24^ During inflammation, oxidative stress promotes the conversion of luminal tryptophan into indoles for AhR activation,^25^ or promote the degradation of quinolinate into NAD+,^26^ protecting the host against inflammation associated epithelial degradation. The effect of the microbiome as an active player in this phenomenon has not yet been described.

To better understand the impact of a long-term, progressive inflammatory condition on the gut–brain axis, tryptophan metabolism and behavior, we studied a mouse model in which the mucin 2 gene is deleted (Muc2 KO; Muc2^−/−^).^27^ The Muc2^−/−^ model of colitis has previously been shown to develop inflammation consistent with chronic colitis and microbial dysbiosis.^28^ ^,^ ^29^ Muc2^−/−^ mice present colonic histopathology and metabolic dysfunction consistent with patients with ulcerative colitis.^30^ ^,^ ^31^ While some behavioral comorbidities have been characterized previously,^32^ associated tryptophan metabolites have not yet been characterized in the Muc2^−/−^ model. We hypothesized that microbiome dysbiosis would correlate with behavioral changes and alterations in neurometabolite concentrations in a chronic model of colitis. Here, we showed that chronic colitis in Muc2^−^^/^^−^ mice was associated with microbiome-driven depletion of intestinal tryptophan and related metabolites, which correlated with behavioral comorbidities, including anxiety and memory dysfunction. Despite chronic inflammation, kynurenine levels were reduced in Muc2^−/−^ mice, which is consistent with TrpNet predictions showing microbial diversion of tryptophan toward indole production and the NAD⁺ salvage pathways, thereby limiting substrate availability for host kynurenine pathway metabolism. These findings suggested that a colitis-associated microbiome depleted host tryptophan stores and may link intestinal inflammation to neurobehavioral outcomes in IBD. Targeting microbiome-mediated tryptophan depletion may therefore provide a therapeutic strategy to address both colitis and its behavioral comorbidities.

## Materials and methods

### Animal models

All experiments were performed at the University of British Columbia–Okanagan under the approval of the Animal Care Committee (Protocol number: A23-0033) in accordance with the guidelines of the Canadian Council on Animal Care.

The study was conducted using specific pathogen-free (SPF) mucin 2 knockout mice (Muc2^−/−^, *n* = 6–8/sex), SPF mucin 2 containing mice (Muc2^+/+^, *n* = 6–8/sex), germ-free (GF) mucin 2 knockout mice (GF Muc2^−/−^, *n* = 3–4/sex) and GF mucin 2 containing mice (GF Muc2^+/+^, *n* = 3–4/sex). Sample size calculations were based on an alpha of 0.05 and beta of 0.2 with a power of 0.8 and resulted in an approximate sample size of 8 mice total. Muc2^−/−^ mice were originally sourced from the Vancouver Gastrointestinal Disease Research Program (B. Vallance lab, University of British Columbia, Vancouver, BC, Canada^27^) and were bred in-house, and backcrossed onto a C57Bl/6 background. Muc2^+/+^ mice, which were originally sourced from Charles River (Montreal, QC, Canada), were similarly bred in-house for at least 4 generations before the start of this study and served as controls for Muc2^−/−^ mice. For the microbiome assessment, SPF Muc2^+/+^ and Muc2^−/−^were bred to create heterozygotes (Muc2^+/^^−^) controls to minimize any potential litter effects. Phenotypically, Muc2^+/^^−^ display mucin 2 within their colon and have not been shown to differ from Muc2^+/+^ mice.^29^ ^,^ ^33^ All the mice had access to sterilized water (non-acidified) and were housed in specific pathogen-free conditions (Ehret Bio A.S. IVC filtered mouse caging, Freiburg, Germany). The mice were maintained on PicoLab® Rodent Diet 5053 throughout this study with free access to food and water. The mice were housed with their littermates in a sex-dependent distribution (2–5 per cage depending on litter size) in a room with 14 h of daylight and 10 h of dark. Litter was shared on a weekly basis across the cages throughout the study to minimize cage effects. Singly housed mice were excluded from this study owing to their potential effects on behavior.

Germ-free mice were sourced from the International Microbiome Centre (University of Calgary, Calgary, AB, Canada), sent in sterile isolators and maintained under GF conditions in an NKP Isotec isolator (Flexible film isolator ISO-Type 1D Isolator, NKP Isotec, Cheshire, UK). The mice were housed in open-top, sex separate cages (3–4 per cage) in a room with 14 h of daylight and 10 h of dark. In addition to open topped caging, litter was shared on a weekly basis across cages throughout the study to minimize cage effects.

### Experimental setup

An experimental outline is shown in Supplemental Figure 1, in brief: Muc2^−/−^ and Muc2^+/+^ mice were reared under SPF or GF conditions for 16 weeks. At 16 weeks of age, the mice underwent behavioral profiling as described below to ensure significant disease severity while reducing the potential for the development of colorectal cancer as previously characterized in this model.^34^ Following completion of the behavioral assessments, the mice were anesthetized using a 2% isoflurane/O_2_ mixture to enable whole blood collection and exsanguination via cardiac puncture. Following exsanguination, confirmation of death occurred via cervical dislocation. Following euthanasia, colonic and brain tissues were collected. The tissues were divided and either flash-frozen in liquid nitrogen, placed in 10% formalin, or placed in RNAprotect (Qiagen, Venlo, Netherlands). The samples were kept in a −80 °C freezer until use.

### Histology

To evaluate tissue pathology, we employed a scoring system as previously described by Bergstrom et al.^35^ Pathological assessment included visually quantifying the amount of inflammatory cell infiltration, epithelial damage, and crypt hyperplasia, and the total score was the cumulative score across all parameters (Supplemental Table 1). Briefly, 5 µm formalin-fixed paraffin-embedded colonic tissue sections were stained with H&E (Wax-it Histology Inc., Vancouver, BC, Canada). The slides were viewed on a Thermo Invitrogen EVOS M5000 phase contrast fluorescence microscope imaging system with both 10× (Olympus UPlanAPO 10×/0.40 OFN26.5) and 20× (Plan Fluor 20x/0.50) lenses. EVOS M5000 imaging system software Life Technologies Corporation, Bothell, Washington, United States). Images were scored (supplemental data on OSF (https://osf.io/9gqp2)) using two independent and blinded scorers.

### RNA extraction and quantitative real-time PCR (qPCR)

Tissues (distal colon and the right hemisphere of the brain to the medulla oblongata following a midsagittal dissection according to Aboghazleh et al.^36^) were removed at the time of euthanasia, placed in RNAprotect (Qiagen, Venlo, Netherlands, Cat. No.: 76104) and stored in a −80 °C freezer until extraction. Tissues were extracted using the Qiagen RNeasy Fibrous Tissue Mini Kit (Qiagen, Venlo, Netherlands, Cat. No.: 74704) according to manufacturer specifications. Total RNA was quantified using a NanoDrop 2000c Spectrophotometer (ThermoScientific, Waltham, Massachusetts, United States), and cDNA was synthesized using BioRad iScript cDNA Synthesis Kit. Quantification of cDNA was performed on a Bio-Rad CFX Manager 2.0 using the Bio-Rad Sso Fast Eva Green Supermix. All primers were synthesized using Integrated DNA Technology, Canada. qPCR primer sequences can be found in the supplemental data (Supplemental Table 2). Gene expression was normalized to eukaryotic elongation factor 2 (*Eef2*) and TATA-binding protein (*Tbp*) mRNA levels according to the Pfaffl method.

### Western blot analysis

Specific pathogen-free Muc2^−/−^ mouse brains were removed at euthanasia. A coronal slice consisting of brain tissue between the confluence of the sinuses and the caudal end of the cerebral cortex was collected and homogenized in radioimmunoprecipitation assay buffer (RIPA, 150 mM sodium chloride, 1% v/v Triton X-100, 0.5% w/v sodium deoxycholate, 50 mM tris(hydroxymethyl) aminomethane (TRIS), pH 8.0) containing a 1% w/v protease inhibitor cocktail. Homogenized brain samples were centrifuged for 4 min at 7000 *g* at 21 °C, and the protein concentration in the supernatant fraction was measured by the bicinchoninic acid assay. Supernatant protein level was determined by bicinchoninic acid assay (Fisher Scientific, Hampton, New Hampshire, United States), performed as per manufacturer instructions. The protein concentrations in all the samples were equalized to 10 mg/ml before dilution in RIPA buffer, and the levels of TNF-α, MCP-1, IFN-γ, GM-CSF, IL-1β, IL-4, IL-6, IL-10, and IL-12 p70 in the supernatant were quantified by Eve Technologies (Calgary, AB, Canada). The supernatant was then concentrated by a factor of two in a Savant™ speedvac concentrator (Fisher Scientific, Hampton, New Hampshire, United States) and used for western blotting analysis.

### Western blot analysis of claudin-5, IBA-1 and GFAP expression

The expression of Claudin-5, ionized calcium-binding adaptor molecule 1 (IBA-1) and glial fibrillary acid protein (GFAP) in mouse brain samples was assessed by immunoblotting. Samples containing 40 µg of protein for Claudin 5 detection, 17 µg of protein for GFAP detection or 27 µg of protein for IBA-1 detection were mixed with an equal volume of loading buffer (4% w/v SDS, 10% v/v 2-mercaptoethanol, 20% v/v glycerol, 0.0004% w/v bromothymol blue) and heated at 90 °C for five min. Sample proteins were then separated by SDS-polyacrylamide gel electrophoresis on 10% polyacrylamide gels. The separated proteins were electroblotted onto a nitrocellulose membrane and immersed in blocking solution (5% w/v skim milk powder in tris-buffered saline-tween (TBS-T)) for one hour. The blocked immunoblots were washed six times with TBS-T over one hour and then incubated overnight with a rabbit anti-actin-β antibody (Santa Cruz Biotechnology, Dallas, TX, United States, Cat No.: #SC-1616-R), and rabbit anti-claudin 5 antibody (Sigma-Aldrich, Oakville, ON, Canada, L6H 6J8, Cat No.: ABT45), a rabbit anti-GFAP antibody (Agilent, Santa Clara, CA, United States, Cat No.: Z033429-2), or rabbit anti-IBA-1 antibody (FUJIFILM Wako Pure Chemical Corporation, Osaka, Japan, Cat No.: 019-19741). All primary antibodies were diluted 1:1000 in blocking solution. After incubation with primary antibodies, the immunoblots were washed again six times with TBS-T over one hour, then incubated for one hour with a aary goat anti-rabbit antibody (Cell Signaling Technology, Whitby, ON, Canada, Cat No.: 7074) diluted 1:1000 in blocking solution. The immunoblots were then washed six times with TBS-T over one hand and then exposed to SuperSignal™ West Pico PLUS enhanced chemiluminescence solution as per manufacturer's instructions. The immunoblots were imaged, and the density of the immunoreactive product was quantified using ImageJ software (1.51). Immunodensity measurements of IBA-1 and GFAP were normalized to those of β-actin.

### Behavioral profiling

Behavioral profiling in this study included tests for anxiety, depression and memory deficit assessments. Mice underwent one of two anxiety-like behavioral assessments (open field or light/dark maze), followed by a depression-like behavioral assessment (tail suspension test), and a memory assessment (novel object recognition test) with a rest period of 24 h between assessments unless otherwise noted below. Mazes were sourced from Maze Engineers (Skokie, IL, USA) or made by Sitka Construction (Kelowna, BC, Canada) and filmed using a GoPro Hero8 at a frame rate of 29.8 frames per second for the duration of the tests. All behavioral profiling was undertaken during the light phase of the day, approximately halfway through the light cycle, to account for circadian rhythm variation. The mice were familiarized with the room before conducting the test. Behavioral tests were completed using the same familiar personnel to reduce handling stress. Videos were assessed using EthoVision XT 17 (Noldus Information Technology, Wageningen, Netherlands) for the open field maze and the novel object. The light/dark maze and tail suspension tests were manually scored with two scorers per test to validate the findings.

### Anxiety-like behavioral assessments

The open field maze (40 cm × 40 cm)^37^ was assessed for the anxiety-like behavioral profile quantified by the amount of time spent in the inner 50% of the maze (s), locomotion parameters including the total distance travelled during the test (cm), the average speed of travel (cm/s^2^), exploratory behaviors indicated by the number of rears (supported and unsupported) during the test, and stress-associated behaviors including grooming episode frequency (#), total grooming episode duration (s), and number of jumps (#) during the trial. The mice were placed in the center of the open field maze, and the handler stepped out of view for the duration of the test. The mice were assessed for a total duration of 10 min, whereupon they were returned to their home cage for 24 h before the next test.

As described by Serchov et al. ^38^ the light/dark maze (light: 25 cm × 40 cm, dark: 17.5 cm × 40 cm) assessed anxiety-like behaviors using the amount of time spent in the light portion of the maze (s), the number of transfers between the light and dark portions of the maze (#) as well as the latency to enter the dark portion of the maze (s). The mice were placed into the light portion of the maze perpendicular to the entrance for the dark portion of the maze. After the test start, the handler stepped out of view for the duration of the test. The mice were assessed for a total duration of 10 min, whereupon they were returned to their home cage for 24 h before the next test.

### Depression-like behavioral assessments

To assess depressive behaviors, the mice underwent the tail suspension test.^39^ ^,^ ^40^ As described by Can et al.^40^ mice were suspended in enclosed spaces and unable to view other mice, with the handler viewing the mice in case of distress during the test.^40^ The mice were suspended for 6 min using a 16 cm long tape attached to their tail with a plastic cuff around the base of the tail to prevent climbing. The time spent immobile was measured with any movement or righting reflex identified as mobile. Following the completion of this test, the mice were returned to their home cage for 24 h before memory-associated behavioral assessments.

### Memory associated behavioral assessments

Associative memory was assessed using the novel object recognition.^41^ ^,^ ^42^ Novel object recognition (NOR) evaluated short and long-term memory retention with exposure to novel and familiar objects. Only SPF mice underwent the novel object recognition test because of the inability to maintain a GF environment over the entire testing period. The mice were habituated in the maze prior to NOR assessment and underwent a 5-min familiarization to acclimatize the mice to two identical familiar objects. Following this familiarization, the mice were returned to their home cage for one hour. The mice that did not spend a minimum of 20 s examining the familiarization objects were disqualified from the remainder of the trial. Following the one-hour rest period, the mice underwent a short-term memory assessment in which an additional object was placed inside the maze in addition to the two familiar objects. Short-term memory assessment was undertaken for 10 min. Mice were monitored for time spent examining the novel object versus the familiar objects. Mice are then returned to their home cages for a 24-h rest period. Following this rest period, the mice re-entered the maze with one familiar object (object seen twice (OST)), the object present for the short-term memory assessment (object seen once (OSO)) and a novel object (NO). Long-term memory assessment was performed for 10 min, and mice were monitored for time spent with the novel object, the short-term memory object and the familiar object.

Both short and long-term memory were assessed based on their object discrimination index. The discrimination index is calculated using the formula PI = (OoO/TT)*100 where OoO is the object of interest (OST, OSO, NO) and is divided by the total time spent with objects during the test (TT). Short-term memory was analyzed using a Welch's *t*-test and analysis of the long-term memory test is completed using a one-way ANOVA with a post hoc Tukey test to compare between the familiar object, short-term memory object, and novel object.

### Serum preparation and GC–MS

Prior to euthanasia, the mice were anesthetized with a 2% isoflurane/O_2_ mixture, and whole blood was collected via cardiac puncture. The whole blood was spun at 1500 x *g* for 15 min to separate the red blood cells and serum. Serum samples were frozen and maintained at −80 °C until use. The samples were analyzed using GC–MS serviced by Innovate Phytoceuticals (Kelowna, BC, Canada).

### Microbiome and predicted pathway analyses

To investigate the association between the Muc2 genotype and microbial features within the context of the study, we reanalyzed our previously published 16S rRNA amplicon sequencing dataset, which included control mice with the Muc2^−/−^ and Muc2^+/^^−^ genotypes, both sexes, and concurrently maintained SPF mice under similar conditions and handling to minimize any temporal or stress-based variation. The raw sequence data and accompanying metadata are publicly available via the Open Science Framework (OSF; DOI 10.17605/OSF.IO/7WYC4). All data preprocessing and the prediction of metabolic functions and pathway abundances based on the MetaCyc database followed the workflow described in the original publication and were not modified for the present study.^33^ In this study, we conducted a hypothesis‑driven analysis of taxa and predicted functions overlapping a curated tryptophan metabolic network, using the TrpNet database (TrpNet‑overlap) and a web platform for microbial tryptophan metabolism (www.trpnet.ca).^43^ Genus-level relative abundances were centered log‑ratio (CLR) transformed before testing. A pooled composite score representing the aggregate CLR-transformed abundance of TrpNet-overlap genera was calculated. Wilcoxon rank‑sum tests were used to compare Muc2^−/−^ versus Muc2^+/^^−^ at both the overall level and within each sex stratum for each TrpNet‑overlap genus and for a pooled score across all such genera. Pathway abundances derived from MetaCyc were compared between Muc2^−/−^ and Muc2^+/^^−^ at the individual pathway level, as well as through a pooled tryptophan-related pathway score. All functional analyses were repeated after stratification by sex. Given the focused, hypothesis‑driven nature of these comparisons, we report raw p‑values without multiple‑testing correction. Analyses were performed in R version 4.5.1 using relevant packages for data handling and statistical testing.

### Early-life nutrient-rich dietary supplementation

Starting at 5 weeks of age, the mice in the supplemented group (Muc2^−/−^ + Sup) received weekly oral gavage of 100 μL of a nutrient-rich mixture of tryptone, yeast extract and ions (Luria bertani broth (LB)) until 8 weeks of age (4 doses total). LB broth was selected because it naturally contains free amino acids rich in glutamic acid as well as tryptophan, as measured at ~3 mg/mL (Supplemental Table 3). This provided approximately 0.3 mg of tryptophan per dose, corresponding to an estimated exposure of ~20 mg/kg/dose, which falls within a physiologically relevant nutritional supplementation range. This approach allowed controlled delivery of physiologically relevant tryptophan as well as other amino acids. Following dosing, the mice returned to the experimental workflow described above.

### Statistical analysis

All the statistics were analyzed using GraphPad Prism (v. 10.6.1, GraphPad, Boston, USA). Data are presented as mean ± standard deviation after testing for normality using a Q–Q plot and were assessed for outliers using Grubb's outlier test. The data were primarily analyzed using either a Welch's *t*-test or one-way ANOVA using a post hoc Tukey multiple comparisons analysis where appropriate. Non-normal data was analyzed using a Mann–Whitney test using Dunn's test to correct for multiple comparisons. An alpha of *p* < 0.05 was defined as significant, and *p* < 0.1 was defined as a trend. Figure panels were assembled in BioRender.

## Results

### The colitis-associated Muc2^−/−^ gut microbiome aggravated colonic damage and elevated indoleamine-2,3-dioxygenase

**Figure 1.**
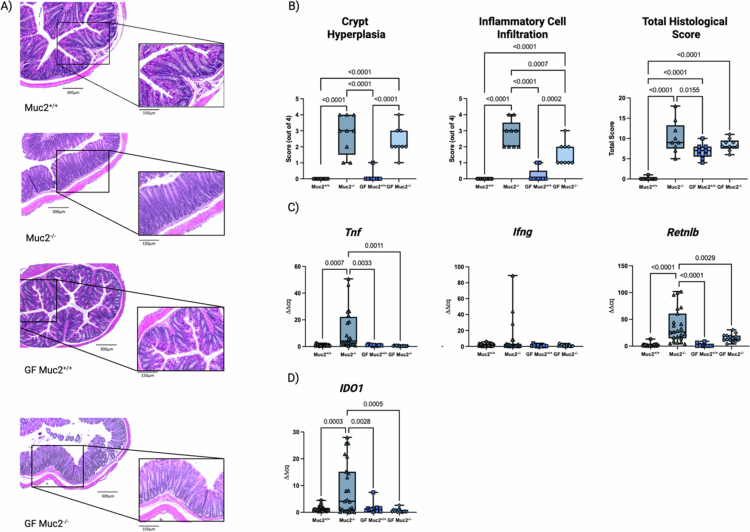
Histological damage increased in *Muc2* ^−/−^ genotypes regardless of microbiome status with subsequent increase in *Retnlb.* Muc2^−/−^ (*n* = 9), Muc2^+/+^ mice (*n* = 9), GF Muc2^+/+^ (*n* = 9), and GF Muc2^−/−^ (*n* = 9) mice were assessed for histological damage with (a) The distal colon was cross-sectioned and underwent H&E staining with representative slides presented. (b) Two blinded scorers assessed inflammatory cell infiltration, crypt hyperplasia, total histological score quantified by Table S2 and *Retnlb*. (c) Gene expression of tumor necrosis factor (*Tnf*) and interferon-gamma (*Ifng*) was measured. *Tnf* was significantly elevated in comparison to all other groups. (d) Gene expression of Indoleamine 2,3-dioxygenase (*Ido1*) was found to be elevated in Muc2^−/−^. Data was analyzed using a one-way ANOVA with post hoc Tukey's multiple comparisons test, with significance set to an alpha set to 0.05 and trends set to 0.1.

To better understand the role of inflammation and epithelial damage in contributing to dysfunction of the microbiome‒gut‒brain axis, we examined histological and inflammatory damage present in both genotypes in both SPF and GF mice. Representative histology for each group is presented in Figure 1A, with the scoring parameters shown (Figure 1B and Supplemental Figure 2). Of note, crypt hyperplasia was elevated in both the SPF and GF Muc2^−/−^ mice compared to both Muc2^+/+^ groups, revealing that this parameter is not dependent on the microbiome. In contrast, the microbiome in Muc2^−/−^ mice is driving inflammatory cell infiltration since there was a lower score in the GF compared to Muc2^−/−^ mice. To verify the microbiome's role in driving inflammation in Muc2^−/−^ mice, we examined colonic inflammatory cytokine genes (Figure 1C). We found elevations of *Tnf* were microbiome dependent. Additionally, Muc2^−/−^ mice displayed a microbiome-dependent elevation of the gene *Retnlb*, which encodes RELM-β, a key indicator in the severity of colitis.^44^ ^,^ ^45^ We also examined the gene encoding indoleamine-2,3-dioxygenase (*Ido1*), the rate-limiting enzyme in the conversion of tryptophan to kynurenine.^46^ We found increased colonic expression of *Ido1* in Muc2^−/−^ mice, indicating further microbiome-dependent inflammatory effects, as no other group displayed this elevation (Figure 1D). Overall, these results reveal that the microbiome is critical for an increased inflammatory response during colitis in the absence of mucin 2.

### Muc2^−/−^ colitis is associated with a dysregulated blood brain barrier and neuroinflammation

**Figure 2.**
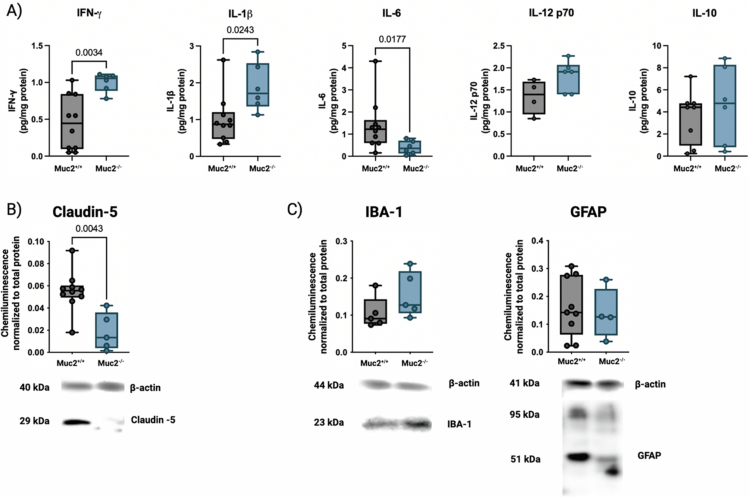
Inflammatory cytokines are increased, and the integrity of the blood-barrier reduced in colitis. Muc2^−/−^ (*n* = 5), Muc2^+/+^ mice (*n* = 5), mice were assessed for blood–brain barrier function and glial activation. (a) Cytokine protein measurements show significant increases in the concentration of IFN-*γ* and IL-1β in the Muc2^−/−^ mice (*p* = 0.0034 and *p* = 0.0243, respectively) compared to Muc2^+/+^ mice. IL-6 protein was significantly decreased in the Muc2^−/−^ mice (*p* = 0.0177). No other significant differences were observed. Western blots of (b) tight-junction barrier protein Claudin-5, and (c) IBA-1 and GFAP activation show decreased concentration of Claudin-5 in the Muc2^−/−^ (*p* = 0.0043) compared to the Muc2^+/+^ mice. No significant differences are observed in IBA-1 or GFAP levels. Data was analyzed using a Welch's *t*-test with significance set to an alpha set to 0.05 and trends set to 0.1.

Colitis, while usually thought of as a local disease, has been associated with other neuroinflammatory conditions.^47^ To understand if the Muc2^−/−^ colitis displayed neuroinflammation we assessed the expression of the cytokines IFN-γ, L-6, IL-1β, IL-12 p70 and IL-10 in the brain of SPF Muc2^−/−^ mice (Figure 2A). Both IFN-γ and IL-1β were increased in Muc2^−/−^ mice. In contrast, IL-6 levels were lower in Muc2^−/−^ mice. Since elevated inflammatory cytokines were present in the brain, we examined if other signs of neuroinflammation were evident. For this, we examined a marker of the blood‒brain barrier, the tight-junction protein claudin-5 (Figure 2B), and markers of glial cell activation, for resident microglia (IBA-1) and astrocytes (GFAP) (Figure 2C). Claudin-5 expression in Muc2^−/−^ mice decreased in relation to the Muc2^+/+^, although IBA-1 and GFAP were not altered with IBA-1 displaying a statistical trend (*p* = 0.0924) only, revealing that while the blood–brain barrier is dysregulated and that there were elevated inflammatory cytokines in the brain, glial activation was evident. Overall, Muc2^−/−^ mice had a dysregulated blood brain barrier and elevated inflammatory cytokines in the brain.

### Altered risk-taking behavior and memory dysfunction are dependent on the microbiome of the Muc2^−/−^mice

**Figure 3.**
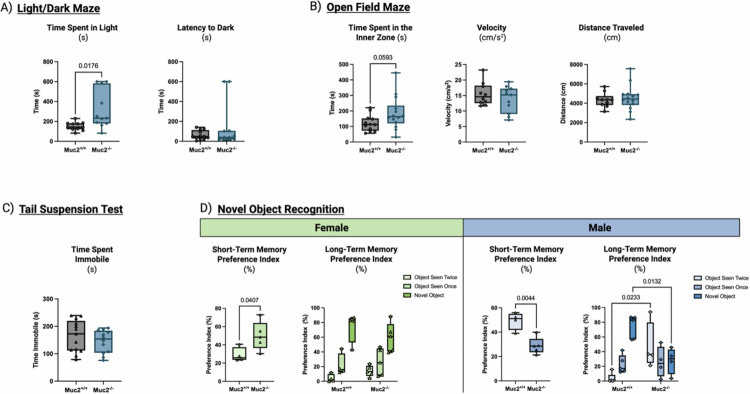
Anxiety and memory deficits are present in a sex-dependent manner for Muc2^−/−^ mice. Muc2^−/−^ (*n* = 9), Muc2^+/+^ mice (*n* = 9), and mice were assessed using behavioral mazes. (a) Light/dark maze (b) open field maze (c) tail-suspension test and (d) novel object recognition test. Significant differences were found in anxiety-like behavior as well as memory dysfunction in a sex-dependent manner. Data was analyzed using a Welch's *t*-test with significance set to an alpha set to 0.05 and trends set to 0.1.

Behavioral comorbidities within IBD have been established for both acute ^48^ and chronic murine models of colitis,^13^ ^,^ ^24^ ^,^ ^49^ the Muc2^−/−^ chronic colitis model.^32^ We examined anxiety-like behaviors, depression-like behaviors and short- and long-term memory dysfunction (Figure 3). Behavioral assessments for colitic Muc2^−/−^ displayed a reduction in anxiety-like behavior in the light/dark maze (*p* = 0.0176) (Figure 3A). There was also a tendency for a modest reduction in anxiety-like behaviors, including time spent in the inner zone (*p* = 0.0593) (Figure 3B). Further assessments showed a sex-differentiated response, indicated by a significantly increased exploratory drive with a higher time in the light for colitic female mice (*p* = 0.0013 compared to female Muc2^+/+^ and *p* = 0.0261 compared to male Muc2^−/−^ mice (Table 1)). Female Muc2^−/−^ mice also showed a significant difference in the time to seek a safe environment (*p* = 0.0208 compared to male Muc2^−/−^) and a tendency of increasing time in the inner zone of the open field maze (*p* = 0.0909) when compared to male Muc2^−/−^ mice (Table 1). The remaining parameters of velocity or the distance travelled during the test were not significantly different. We utilized the tail-suspension test to examine the mice for depressive-like behaviors but found similar inactive periods. Finally, the symptoms of memory dysfunction were assessed in SPF mice. Both short and long-term memory were affected (Figure 3D). Short-term memory was sex-differentiated between Muc2^−/−^ mice, with males spending significantly less time with the novel object (*p* = 0.0044), whereas the female Muc2^−/−^ mice showed a higher preference for the novel object (*p* = 0.0407), reinforcing the increased exploratory behavior found in the light/dark maze. Long-term memory in male mice shows evidence of significant disorganization in contrast to the male Muc2^+/+^, with a majority of their focus being on objects seen previously (*p* = 0.0233 for the object seen twice, *p* = 0.0132 for the novel object). Female mice with colitis display a typical response of focus on novel objects. In conclusion, SPF mice display more risk-taking behavior and memory deficits during colitis.

**Table 1.** Sex-specific differences in behavioral tests separated by genotype.

Maze	Parameter	Genotype/sex	Mean (s) (±SD)	Genotype/sex	Mean (s) (±SD)	*p*-value
Light/dark	Time in light	Muc2^−/−^ Female	418.364 (±246.395)	Muc2^−/−^ Male	233.390 (±81.654)	0.0261
		Muc2^+/+^ Female	127.395 (±33.040)	Muc2^−/−^ Female	418.364 (±246.395)	0.0013
	Latency to enter the dark	Muc2^−/−^ Female	266.596 (±306.524)	Muc2^−/−^ Male	47.043 (±32.244)	0.0208
		Muc2^+/+^ Female	33.927 (±17.878)	Muc2^−/−^ Female	266.596 (±306.524)	0.0151
Open field	Time in inner zone	Muc2^−/−^ Female	126.833 (±68.385)	Muc2^−/−^ Male	189.072 (±75.973)	0.0909

Muc2^−/−^ (*n *= 9), Muc2^+/+^ (*n *= 9), mice were assessed using behavioral mazes. (a) Light/dark maze (b) open field maze. Significant differences were found in anxiety-like behavior as well as memory dysfunction in a sex-dependent manner. Data was analyzed using a one-way ANOVA with post hoc Tukey multiple comparisons test with significance set to an alpha set to 0.05 and trends set to 0.1.

### The absence of a colitic microbiome resolves associated behavioral dysfunction

**Figure 4.**
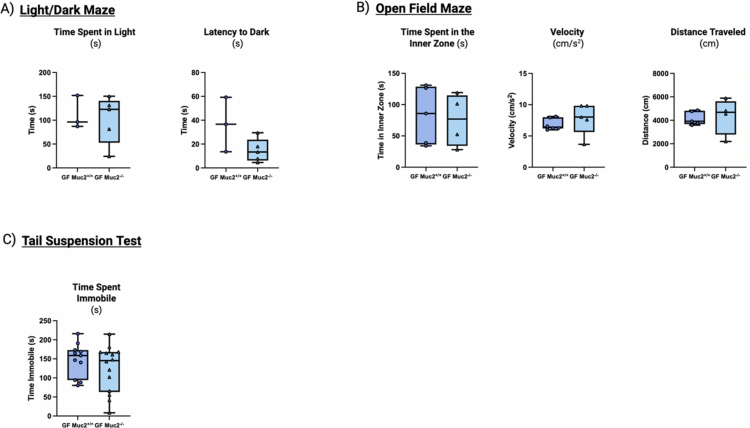
No behavioral differences were observed in mice without a microbiome. Germ-free (GF) Muc2^+/+^ (*n* = 9) and GF Muc2^−/−^ (*n* = 9) mice were assessed using behavioral mazes. (a) Light/dark maze, (b) open field maze and (c) tail-suspension test. No significant differences were found in any of the behavioral test. Data was analyzed using a Welch's *t*-test with significance set to an alpha set to 0.05 and trends set to 0.1.

As a dysbiotic microbiome is predicted to be a driver of colitis,^50^ and the inflammation associated with colitis has been shown to modify behaviors,^24^ we examined behavioral tests in GF mice to elucidate the role of the microbiome in the microbiome‒gut‒brain axis. While germ-free mice have documented behavioral abnormalities in anxiety-like behaviors and depressive-like behaviors,^51^ ^,^ ^52^ this study worked to compare the behavioral profiles across microbiome status and inflammatory profile within the same genotype. As noted in Figure 1C, the inflammatory cytokine profiles *and Ido1* enzyme gene expression in the GF mice were consistent with those in the Muc2^+/+^ mice, indicating no colonic inflammation. Germ-free Muc2^+/+^ and Muc2^−/−^ underwent behavioral profiling to elucidate the role of the microbiome in behavior. Anxiety-like behavior assessments included both light/dark maze and open field assessments (Figure 4A and B). All anxiety-like behavioral patterns were normalized in the chronic colitis model and comparable to those in Muc2^+/+^ mice. Similarly, when examined for depressive-like behavior, (Figure 4C) periods of inactivity were found to be similar across the genotypes (*p* = 0.3022). A comparison between all behavior tests across microbiome status is shown in Supplemental Figure 3. In conclusion, the GF status eliminated the behavioral changes associated with colitis in the Muc2^−/−^ model. Overall, these results confirm the role of the microbiome in driving behavioral comorbidities during colitis.

### Presence of a colitis-associated microbiome and concurrent inflammation contribute to tryptophan depletion

**Figure 5.**
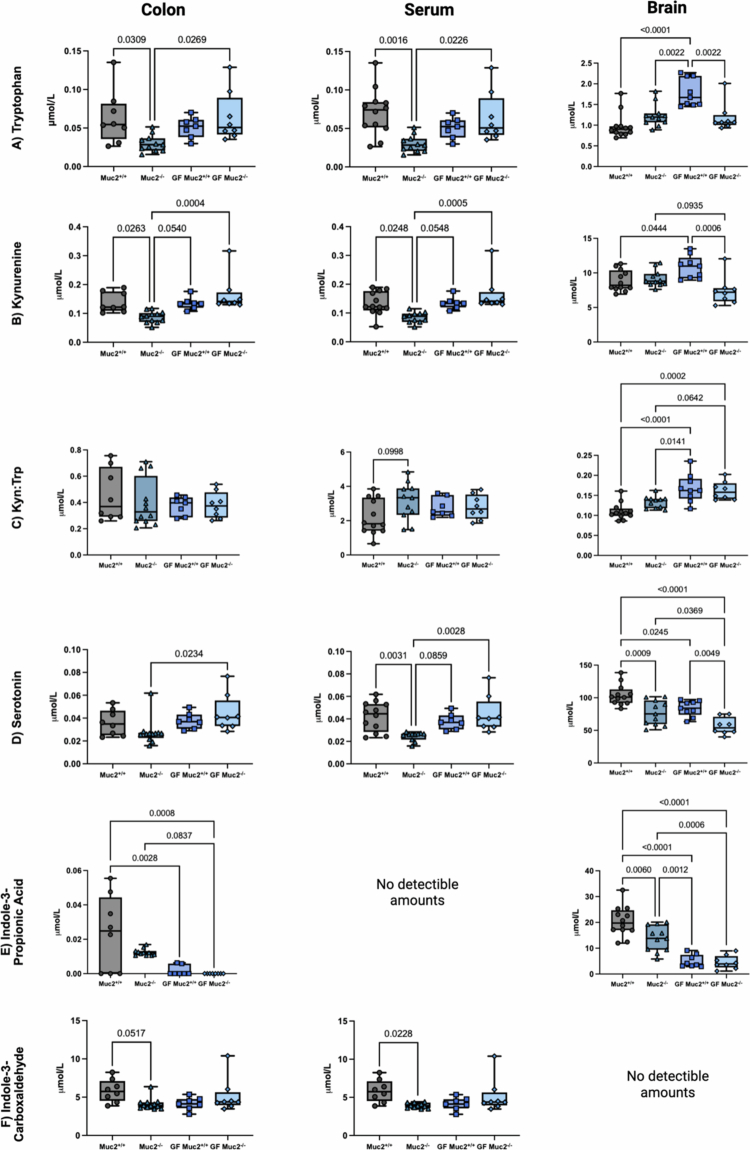
Tryptophan and its metabolites were depleted in SPF but not GF mice with chronic colitis. Concentrations of tryptophan, kynurenine, serotonin, indole-3-propionic acid, and indole-3-carboxyaldehyde were assessed using GC–MS in the colon, serum and brain of Muc2^+/+^ mice (*n* = 12), Muc2^−/−^ (*n* = 11), GF Muc2^+/+^ (*n* = 7), and GF Muc2^−/−^ (*n* = 8) mice. Muc2^−/−^ showed significant depletions in colonic tissue and serum across tryptophan and metabolites. Data was analyzed using a one-way ANOVA with post hoc Tukey's multiple comparisons test with significance set to an alpha set to 0.05 and trends set to 0.1.

Indoleamine 2,3-dioxygenase (IDO) is responsible for converting ~90% of available tryptophan to kynurenine, with other metabolites, serotonin and indoles, being produced from the remaining 10%.^53^ Since colonic IDO-1 impacts tryptophan metabolism,^15^ we examined tryptophan, kynurenine, serotonin, indole-3-proprionic acid and indole-3-carboxaldehyde concentrations across the colonic and brain tissues and serum (Figure 5). There was a striking depletion of tryptophan in Muc2^−/−^ mice compared to Muc2^+/+^(*p* = 0.0309 for colonic tissue, *p* = 0.0016 for serum) (Figure 5A). This depletion was subject to the presence of a microbiome, given that there was a significant decrease compared to GF Muc2^−/−^ (*p* = 0.0269 for colonic tissue and *p* = 0.0226 for serum). Within Muc2^+/+^ mice, tryptophan was also found to display sex-specific differences (Table 2) in both the colon and serum. These within-group differences were not exhibited by any other genotype or microbiome status. The reduction in tryptophan concentrations in the Muc2^−/−^ was then examined for the effects on the downstream products, kynurenine (Figure 5B). Consistent with tryptophan concentrations, kynurenine was found to be significantly lower in Muc2^−/−^ mice compared to the Muc2^+/+^ mice (kynurenine, colon: *p* = 0.0263 compared to Muc2^+/+^, kynurenine, serum: *p* = 0.0248 compared to Muc2^+/+^). We also see consistent, microbiome-based depletion of the tryptophan metabolic pathway in Muc2^−/−^ mice in the colonic tissue and serum when compared to its GF equivalent (kynurenine, colon: *p* = 0.0004 compared to GF Muc2^−/−^, kynurenine, serum: *p* = 0.0005 compared to GF Muc2^−/−^). In contrast to those in the colon and serum, the stool concentrations (Supplemental Figure 4) of kynurenine were not different across genotypes and microbiome status, potentially explained by epithelial release of previously metabolized products, as previously noted.^54^ To better understand the metabolism and conversion of tryptophan within GI tissues, we examined the ratio between tryptophan and kynurenine across all treatments (Figure 5C). Within the colonic tissues, there were no differences in the metabolism of tryptophan to kynurenine.

**Table 2.** Alterations in tryptophan metabolite concentrations in the colon and serum separated by sex and genotype.

Tissue	Metabolite	Genotype/sex	Mean (μmol/L) ( ± SD)	Genotype/sex	Mean (μmol/L) (±SD)	*p*-value
Colon	Tryptophan	Muc2^+/+^ Female	0.091 (±0.041)	Muc2^+/+^ Male	0.047 (±0.019)	0.0127
Serum	Tryptophan	Muc2^+/+^ Female	0.090 (±0.028)	Muc2^+/+^ Male	0.052 (±0.021)	0.0066

Concentration of tryptophan was assessed using GC-MS in the colon, and serum of Muc2^+/+^ mice (*n* = 12), Muc2^−/−^ (*n* = 11), GF Muc2^+/+^ (*n* = 7), and GF Muc2^−/−^ (*n* = 8) mice. Sex-dependent responses are indicated with their mean ± SD. Significant differences and associated *p*-values are indicated. Data was analyzed using a one-way ANOVA with post hoc Tukey's multiple comparisons test, with significance set to an alpha set to 0.05 and trends set to 0.1.

Influenced by inflammation and microbial modulation,^55^ serotonin was also examined in the tissues (Figure 5D). Within the colon, while decreased in concentration, the only significant difference was found within the Muc2^−/−^ genotype. Colonic concentrations of serotonin in SPF mice displayed a significant decrease compared to their GF equivalent (*p* = 0.0234). The inflammation and the microbiome contribute to a decrease in relation to both Muc2^+/+^ mice (*p* = 0.0031) and GF Muc2^−/−^ (*p* = 0.0028). Stool serotonin concentrations remained stable across the microbiome status and all genotypes (Supplemental Figure 4).

Finally, we examined two indoles involved in epithelial barrier maintenance: indole-3-propionic acid (IPA) and indole-3-carboxaldehyde (I3C) (Figure 5E and F). Consistent with the healthy epithelial barrier shown in Figure 1A, we found that IPA was elevated in Muc2^+/+^ mice when compared to GF Muc2^−/−^ mice (*p* = 0.0028). Notably, there was also a trend observed between our Muc2^−/−^ groups (*p* = 0.0837), with SPF mice showing elevated levels of IPA compared to the GF Muc2^−/−^. No detectible levels of IPA were present in the serum. When examining the stool, IPA was present despite the GF status or mouse genotype and can be explained by the concentration within the diet itself. Colonic and serum concentrations of I3C were found to be different in those mice with a microbiome (Muc2^+/+^ and Muc2^−/−^) only (*p* = 0.0517 and 0.0228, respectively). No detectable levels of I3C were present within the stool. Overall, tryptophan metabolism is dysregulated during colitis, with a marked depletion of tryptophan and associated metabolites potentially associated with a dysbiotic microbiome or influenced by inflammation.

### Cerebral requirements and microbial feedback regulate tryptophan and metabolite concentrations in the brain

To better understand the effects of tryptophan depletion, we investigated the concentrations of tryptophan and subsequent metabolites within brain tissue (Figure 5). It has been previously observed that GF mice have alterations in metabolite concentrations when compared to their SPF counterparts.^17^ ^,^ ^56^ In our study, we observed tryptophan and kynurenine were elevated in the brains of GF Muc2^+/+^ mice (*p* = < 0.0001 compared to Muc2^+/+^ and GF Muc2^−/−^ for tryptophan and *p* = 0.0354 compared to Muc2^+/+^ and *p* = 0.0005 compared to GF Muc2^−/−^ Figure 5A and B). Importantly, the GF Muc2^−/−^ mice did not show this elevation within the brain; rather, they showed levels comparable to those of the genotypes that had a microbiome, dysbiotic or not. It is currently unclear why this reduction in tryptophan and kynurenine levels within the GF Muc2^−/−^ was observed when compared to the GF Muc2^+/+^ mice; however, alterations in the gut epithelial barrier and a lack of a microbiome may impact the ability of the brain to regulate tryptophan uptake and metabolism through the blood‒brain barrier. Within the brain, genotype- and sex-specific differences were also observed in the concentrations of tryptophan and serotonin. Muc2^+/+^ female mice, regardless of their microbiome status, presented significantly higher concentrations of tryptophan and serotonin (Table 3). However, this was not observed in the tryptophan concentrations for the male Muc2^−/−^ mice, which had higher concentrations of serotonin within the brain than their female counterparts (*p* = 0.0457) (Table 4).

**Table 3.** Brain tryptophan metabolite concentrations separated by sex and genotype.

Tissue	Metabolite	Genotype/sex	Mean (μmol/L) (±SD)	Genotype/sex	Mean (μmol/L) (±SD)	*p*-value
Brain	Tryptophan	Muc2^+/+^ Female	1.146 (±0.372)	Muc2^+/+^ Male	0.823 (±0.096)	0.0359
		Muc2^+/+^ Male	0.823 (±0.096)	Muc2^−/−^ Male	1.381 (±0.279)	0.0034
		Muc2^−/−^ Female	1.060 (±0.147)	Muc2^−/−^ Male	1.381 (±0.279)	0.0457
		GF Muc2^+/+^ Female	2.216 (±0.046)	GF Muc2^+/+^ Male	1.572 (±0.135)	0.0012
	Serotonin	Muc2^−/−^ Female	94.876 (±6.760)	Muc2^−/−^ Male	61.776 (±8.610)	<0.0001

Concentration of tryptophan and serotonin were assessed using GC–MS in the brain of Muc2^+/+^ mice (*n* = 12), Muc2^−/−^ (*n* = 11), GF Muc2^+/+^ (*n* = 7), and GF Muc2^−/−^ (*n* = 8) mice. Sex-dependent responses are indicated with their mean ± SD. Significant differences and associated *p*-values are indicated. Data was analyzed using a one-way ANOVA with post hoc Tukey's multiple comparisons test with significance set to an alpha set to 0.05 and trends set to 0.1.

**Table 4.** TrpNet highlights indole pathway through microbiome genera overlap.

Pathway	Cutoff	n_TrpNet_genera_pathway_all	n_TrpNet_genera_pathway_cutoff	n_microbiome_genera_overlap
Indole	1	586	12	0
Indole	0.9	586	470	17
Indole	0.8	586	492	17
Indole	0.7	586	517	17
Indole	0.6	586	549	20
Indole	0.5	586	554	21
Kynurenine	1	586	9	0
Kynurenine	0.9	586	20	0
Kynurenine	0.8	586	37	0
Kynurenine	0.7	586	44	0
Kynurenine	0.6	586	49	0
Kynurenine	0.5	586	56	1
Tryptamine	1	586	5	0
Tryptamine	0.9	586	6	0
Tryptamine	0.8	586	32	0
Tryptamine	0.7	586	35	0
Tryptamine	0.6	586	44	0
Tryptamine	0.5	586	49	1

Previously published 16S rRNA amplicon sequencing dataset which included Muc2^+/−^ and Muc2^−/−^ maintained under the same study conditions (OSF; DOI 10.17605/OSF.IO/7WYC4) conducted a hypothesis-driven analysis of taxa and predicted functions through the TrpNet database. Pooled composite score representing the aggregate CLR-transformed abundance of TrpNet-overlap genera was calculated.

Despite the lower concentrations, the kyn:trp ratio within the brain was elevated in the GF mice when compared to SPF mice (Figure 5C). GF Muc2^+/+^ and GF Muc2^−/−^ were found to be higher than both of their comparable SPF genotypes (GF Muc2^+/+^ *p* = < 0.0001 compared to Muc2^+/+^, GF Muc2^−/−^: *p* = 0.0642 compared to Muc2^−/−^). Within microbiome status, there were no significant differences between kyn:trp ratio across genotypes. Importantly, the levels of serotonin in the brain do not show similar trends to those of tryptophan and kynurenine (Figure 5D). Despite tryptophan and kynurenine being elevated in the brain for the GF Muc2^+/+^ mice, serotonin shows depletion in all other groups compared to Muc2^+/+^ mice. In particular, the GF Muc2^−/−^, despite not showing physiological depletion outside of the brain, showed a significant decrease when compared to its GF Muc2^+/+^ counterpart (*p* = 0.0049). When comparing the GF and Muc2^−/−^ mice, there is a notable depletion (*p* = 0.0369) in serotonin, suggesting regulation of serotonin within the brain is partially microbiome dependent. However, microbiome regulation is not entirely responsible for this depletion, as Muc2^−/−^ mice show significantly less serotonin than Muc2^+/+^ mice (*p* = 0.0009), despite having a microbiome. Overall, microbial feedback regulates tryptophan metabolites in the brain.

### The colitis-associated microbiome in the Muc2^−/−^ predicts indole production and NAD + salvage pathways

**Figure 6.**
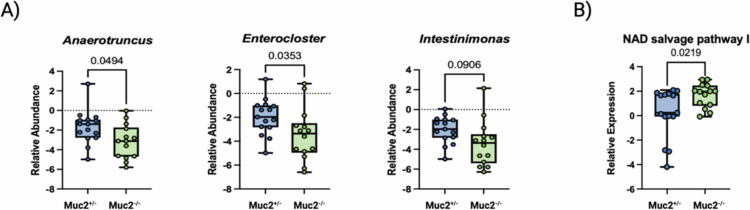
Muc2^−/−^ microbiome shows depletion in key tryptophan metabolizing bacterial species while enriching the NAD + salvage pathway. Muc2^+/^^−^ mice (*n* = 12) and Muc2^−/−^ (*n* = 11) TrpNet comparative analysis was completed from previously published microbiome data. (a) Muc2^−/−^ showed significant depletions in *Anaerotruncus* (*p* = 0.0494), *Enterocloster* (*p* = 0.0353) and trending depletion in *Intestinimonas*. (b) NAD + salvage pathway I was significantly increased in the Muc2^−/−^ mice when compared to Muc2^+/^^−^ (*p* = 0.0219). Data was analyzed using a Wilcoxon rank-sum tests, with significance set to an alpha set to 0.05 and trends set to 0.1.

To better understand whether genotype-associated microbial features, we examined taxa and predicted tryptophan-related pathways in Muc2^+/^^−^ and Muc2^−/−^ mice. The pooled tryptophan-related genera were not different between genotypes (*p* = 0.326). Similarly, the predicted tryptophan-related functional potential did not differ at the pooled level (*p* = 0.539). Both suggest no genotype-associated difference in tryptophan-related genus and predicted pathway capacity between Muc2^+/^^−^ and Muc2^−/−^. Despite the absence of pooled differences, select TrpNet-overlap genera differed between genotypes. *Anaerotruncus* (*p* = 0.0382), *Enterocloster* (*p* = 0.0343), and *Intestinimonas* (*p* = 0.0307) were reduced in Muc2^−/−^ mice compared to Muc2^+/^^−^ mice (Figure 6A). At the functional level, we observed an increase in the predicted NAD + salvage pathway I (Figure 6B) in Muc2^−/−^ mice (*p* = 0.0219). Together, these findings suggest that genotype-associated differences were evident at the level of selected genera and one predicted pathway rather than as a coordinated shift in pooled tryptophan-related microbial features.

When stratified by sex, genotype-associated differences remained genus-specific, although the taxa contributing to the signal varied by sex. In females, *Anaerotruncus* (*p* = 0.0081) and *Intestinimonas* (*p* = 0.0199) were decreased in Muc2^−/−^ mice, whereas *Parasutterella* was increased (*p* = 0.0343). In males, *Mucispirillum* was elevated in Muc2^−/−^ mice (*p* = 0.0124), and *Prevotella* was decreased (*p* = 0.0268). The pooled tryptophan-related genera did not show significant differences in either females (*p* = 0.432) or males (*p* = 0.459), and the pooled tryptophan-related pathways also remained non-significant in females (*p* = 0.915) and males (*p* = 0.772) (supplemental data on OSF (https://osf.io/9gqp2)). Several pathways showed trends, including serotonin degradation and the NAD salvage pathway I in females (*p* = 0.0567 for both) and NAD biosynthesis I in males (*p* = 0.0538). Overall, these data support genotype-associated differences in selected microbial taxa and predicted functional features but do not indicate a broad global shift in pooled tryptophan-related microbial capacity.

### Early-life nutrient rich dietary supplementation results in partial phenotype normalization

**Figure 7.**
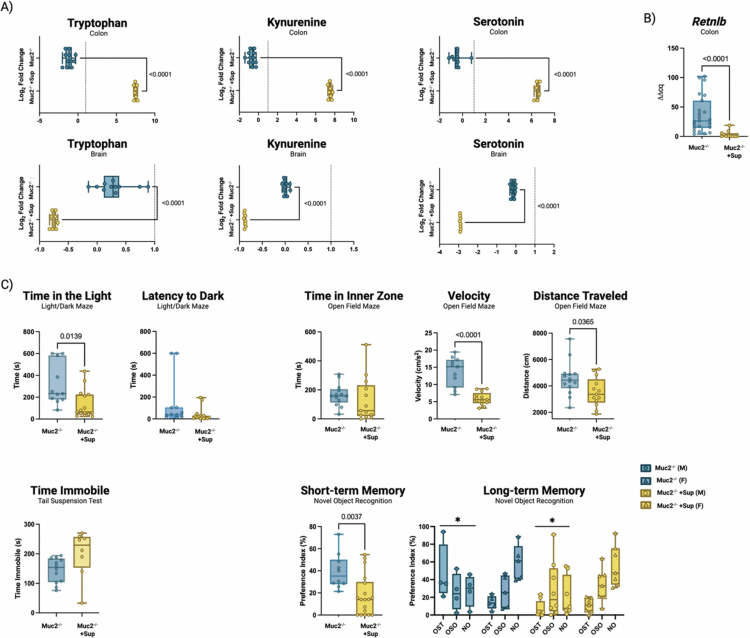
Tryptophan and metabolite supplementation, does not ameliorate all the behavioral comorbidities. (a) Tryptophan, kynurenine and serotonin were measured in colonic and brain tissue and represented as the fold change for both Muc2^−/−^ and Muc2^−/−^ + Sup compared to Muc2^+/+^ mice. Significant increases were observed in the colonic tissue (*p* < 0.0001) for all metabolites. Brain tissue for Muc2^−/−^ + Sup displayed a significant decrease in metabolite concentration in relation to Muc2^−/−^ tissue (*p* < 0.0001). (b) *Retnlb* gene expression levels in the Muc2^−/−^ + Sup group (*n* = 11) were significantly decreased when compared to previously reported Muc2^−/−^ *Retnlb* (shaded boxes from Figure 1) (*p* < 0.0001). (c) Muc2^−/−^ (*n* = 9), previously reported in Figure 3 (shaded box), and Muc2^−/−^ + Sup mice were assessed using behavioral mazes (a) Light/dark maze. (b) Open field maze. (c) tail-suspension test and (d) novel object recognition test. Assessments showed a normalization of velocity and the time spent in the light. Short-term memory showed a significant decrease in novel object preference, and long-term memory showed that early-life supplementation was unable to ameliorate sex-dependent dysfunction (marked by * for both Muc2^−/−^ males and Muc2^−/−^ + Sup males). Data were analyzed using a Welch's *t*-test or with a one-way ANOVA where relevant, with significance set to an alpha set to 0.05 and trends set to 0.1.

Attempting to recover the effects from the tryptophan and metabolite depletion in the Muc2^−/−^, we tested the effect of juvenile supplementation with a rich source of free amino acids, including tryptophan, on behavioral patterns. Supplementation restored tryptophan, kynurenine and serotonin in the colon tissues (Figure 7A; *p* < 0.0001) but not in the brain. The colitic severity was also reduced with early-life supplementation, resulting in a reduction of *Retnlb* (*p* < 0.0001), a sign of severe inflammation in untreated Muc2^−/−^ mice (Figure 7B). Evaluation of behaviors at week 16 (Figure 7C) revealed decreases in locomotion in the open field maze for both velocity (*p* < 0.0001 when compared to reported Muc2^−/−^ values from Figure 3 (shaded)) and a decrease in distance travelled (*p* = 0.0365 compared to Muc2). Assessment of anxiety-like behaviors highlighted a normalization in the amount of time spent in the light for Muc2^−/−^ + Sup (*p* = 0.0139 compared to Muc2^−/−^). Depression-like behavior was unchanged (*p* = 0.1295). Supplementation resulted in short -term memory dysfunction (*p* = 0.0037 when compared to Muc2^−/−^) with mice spending increased time with the familiar object, foregoing the novel object. Long-term memory dysfunction in the male group was not restored with supplementation. The marked results (*p < 0.05, Figure 7) highlight that the dysfunctional pattern preferentially focusing on either the familiar or short-term object rather than the novel long-term memory object. Overall, a nutrient-rich bolus introduced in early life had a partial effect on the gut‒brain axis during colitis.

## Discussion

IBD is increasingly associated with neurobehavioral comorbidities, including anxiety, depression, and cognitive dysfunction. However, the mechanisms linking intestinal inflammation to behavioral outcomes remain incompletely understood. In this study, we demonstrated that chronic colitis in the Muc2-deficient mouse model is associated with microbiome-dependent depletion of intestinal tryptophan and related neurometabolites, accompanied by behavioral alterations, including increased risk-taking behavior and memory dysfunction. Importantly, these behavioral phenotypes were absent in GF mice, indicating that the microbiome plays a central role in linking intestinal inflammation to behavioral outcomes. We also demonstrate that behavioral comorbidities can be improved only partially with dietary supplementation using a diet rich in free amino acids.

Our findings support a model in which loss of the mucin-2 barrier promotes microbiome-driven inflammation that alters host tryptophan metabolism. Consistent with previous studies showing that chronic inflammation drives tryptophan catabolism,^16^ ^,^ ^24^ we observed depletion of tryptophan and several downstream metabolites in the colon and serum of colitic mice. This was accompanied by increased gene expression of *Ido1*, a key enzymatic regulator of tryptophan metabolism that is induced by inflammatory cytokines such as TNF-α and IFN-γ.^57^ These results are consistent with previous work demonstrating that inflammatory signaling promotes the diversion of tryptophan metabolism during chronic disease states.^58^

In addition to host-driven metabolic changes, our results suggest that the dysbiotic microbiome contributes to altered tryptophan utilization. Microbiome functional analysis indicated increased representation of microbial pathways associated with indole production and NAD⁺ salvage metabolism. These pathways are known to play roles in oxidative stress responses and host–microbe signaling.^58‐60^ Shifts in specific bacterial taxa, including reductions in psychobiotic bacteria *Anaerotruncus*, *Enterocloster*, and *Intestinimonas*, further suggest that chronic inflammation reshapes the microbial community in ways that alter neurometabolite availability.^61^ Together, these findings indicate that microbial metabolism may compete with or compensate for host pathways for available tryptophan during chronic inflammation.

Despite significant depletion of tryptophan metabolites in peripheral tissues, concentrations of tryptophan and kynurenine within the brain remained relatively stable. This suggests that the central nervous system may maintain local regulation of these metabolites, potentially through blood–brain barrier transport mechanisms or local metabolic pathways. However, serotonin concentrations in the brain were reduced in colitis mice, suggesting that specific branches of the tryptophan pathway may remain vulnerable to systemic metabolic disruption. Given the role of serotonin in mood regulation and cognitive processes,^62^ ^,^ ^63^ this reduction may contribute to the behavioral phenotypes observed in this study.

Behavioral analysis revealed notable sex-dependent differences in the response to chronic colitis. Female mice exhibited reduced anxiety-like behaviors characterized by increased exploratory activity, while male mice displayed deficits in memory performance. Similar alterations in exploratory behavior have been reported in previous studies of chronic intestinal inflammation and microbiome perturbation,^32^ suggesting that changes in risk-taking behavior may represent an adaptive or compensatory response to chronic physiological stress. Importantly, these behavioral changes were not observed in GF animals, further supporting the role of microbiome-dependent signaling in modulating the gut–brain axis.

To explore whether the restoration of tryptophan availability could improve behavioral outcomes, we performed an early-life supplementation experiment. Nutrient-rich supplementation increased the colonic concentrations of tryptophan and downstream metabolites and reduced the expression of *Retnlb* but did not fully normalize the behavioral phenotypes or brain metabolite levels. These findings suggest that while tryptophan depletion contributes to the metabolic disturbances observed during colitis, additional factors, including persistent inflammation and microbiome restructuring, likely play important roles in driving behavioral dysfunction.

Although comparisons between SPF and germ-free mice implicate the microbiome in the observed metabolic and behavioral phenotypes, our study was limited by a lack of direct microbiome manipulation experiments to establish causality. In addition, this study focused primarily on tryptophan metabolism and did not assess other microbiome-derived metabolites, including short-chain fatty acids and glutamate, that may contribute to gut–brain axis signaling. This study was also limited by targeting behavioral assessments for a single time point during established colitis, limiting the ability to determine temporal relationships between disease progression, metabolic changes, and behavioral outcomes, and with the smaller sample sizes, particularly for germ-free behavioral assessments. Finally, while sex-specific behavioral differences were observed, the mechanisms underlying these differences remain to be fully elucidated.

Collectively, our results support a model in which chronic colitis disrupts the intestinal mucus barrier, leading to microbiome dysbiosis and inflammatory signaling that alters tryptophan metabolism and gut–brain communication. These changes are associated with behavioral alterations that are dependent on the presence of the microbiome. This work highlights the importance of microbiome-mediated metabolic pathways in shaping host physiology during chronic inflammatory disease and suggests that therapeutic strategies targeting microbiome–metabolite interactions may provide new opportunities for addressing the behavioral comorbidities associated with IBD.

## Supplementary Material

Supplementary Material KGMI_A_2689121.zip

Supplementary Material Supplementary_tables.xlsx

## Data Availability

Data can be found on Open Science Framework (https://osf.io/9gqp2).
